# Competition Effects during Femtosecond Laser Induced Element Redistribution in Ba- and La-Migration Based Laser Written Waveguides

**DOI:** 10.3390/ma14123185

**Published:** 2021-06-09

**Authors:** Manuel Macias-Montero, Pedro Moreno-Zárate, Francisco Muñoz, Belén Sotillo, Marina Garcia-Pardo, Rosalía Serna, Paloma Fernandez, Javier Solis

**Affiliations:** 1 Laser Processing Group, Institute of Optics (IO, CSIC), Serrano 121, 28006 Madrid, Spain; manuel.macias@csic.es (M.M.-M.); r.serna@io.cfmac.csic.es (R.S.); 2 Electronic Engineering Department, National Technological Institute of Mexico, Campus Acatlan de Osorio, Carretera Acatlan—San Juan Ixcaquistla k.m. 5.5, Acatlan de Osorio 74949, Mexico; pemzamx@gmail.com; 3 Institute of Ceramics and Glass (ICV, CSIC), Kelsen 5, 28049 Madrid, Spain; fmunoz@icv.csic.es; 4 Department of Materials Physics, Faculty of Physics, Complutense University of Madrid, 28040 Madrid, Spain; bsotillo@fis.ucm.es (B.S.); arana@fis.ucm.es (P.F.)

**Keywords:** fs-laser writing, waveguides, element redistribution, Soret effect, diffusion competition effects

## Abstract

Fs-laser induced element redistribution (FLIER) has been a subject of intensive research in recent years. Its application to various types of glasses has already resulted in the production of efficient optical waveguides, tappers, amplifiers and lasers. Most of the work reported on FLIER-based waveguides refers to structures produced by the cross-migration of alkali (Na, K) and lanthanides (mostly La). The latter elements act as refractive index carrying elements. Herein, we report the production of Ba-based, FLIER-waveguides in phosphate glass with an index contrast > 10^−2^. Phosphate glasses modified with the same amount of Na_2_O and K_2_O, and variable amounts of BaO and/or La_2_O_3_ were used to produce the FLIER-waveguides with Ba and or La acting as index carriers. Ba-only modified glasses show a waveguide writing threshold and light guiding performance comparable to that of La-based structures. However, mixed Ba-La glasses show a much higher element migration threshold, and much smaller compositionally modified regions. This behavior is consistent with a competition effect in the cross-migration of both elements (Ba and La) against the alkalis. Such an effect can be applied to inhibit undesired element redistribution effects in fs-laser processing applications in multicomponent glasses.

## 1. Introduction

The modification of the local composition of glasses irradiated underneath the surface with focused femtosecond laser beams has been studied for a long time [1,2]. This effect has recently gained considerable attention [3] due to its successful use for the production of several different high-performance photonic devices [4,5,6]. Femtosecond-induced element redistribution (FLIER) can be considered as a universal phenomenon in multicomponent glasses subjected to subsurface, high repetition rate, fs-laser excitation. This phenomenon can be understood in terms of the thermo-diffusion processes (i.e., the Ludwig–Soret effect [7]) associated with the chemical potential imbalance induced in the heated glass volume. The glass compositional modification upon FLIER mimics the elemental thermal diffusion occurring in basaltic liquids (silicates) in the Earth’s mantle, but on a much shorter time scale (10^8^ times faster) and over a well-defined micrometric volume [8]. The focal volume shape, local thermal gradients and heating-cooling cycles thus strongly condition the final result of the FLIER process [3,8]. Its effects can be particularly relevant when both relatively heavy (e.g., lanthanides) and light elements (e.g., alkali) are present in the glass composition. In spite of this, FLIER effects have apparently been overlooked in the past, in many occasions, in the discussion of the origin of the refractive index contrast [9,10] in fs-laser-written waveguides [11].

Thermo-diffusion involves several parameters including thermal and stress gradients, viscosity and its temperature dependence, multiple diffusion coefficients [3], etc. At the end of the day, these parameters condition the local chemical potential of the different glass constituents [12]. Indeed, the prediction of the sign and magnitude of the Soret coefficient of a given component in a multicomponent mixture is still an open problem that requires to consider microscopic parameters and numerical modeling [13]. Still, FLIER can certainly be controlled in practice [14,15] and exploited for producing high-performance photonic devices, as indicated above. Even more, one of the key features of FLIER-based fs-laser writing for photonic applications arises from the potential of ad hoc glass compositional design [4,16]. A given glass composition can be slightly modified by adding small amounts of a modifier made of a relatively heavy ion-oxide (that will act as refractive index carrier) and fast diffusing and lighter (typically alkali) ion-oxide modifier that counter-migrates the index carrier. This gives rise to the formation of regions with increased refractive index and light guiding capabilities. As a matter of fact, by using the electronic polarizabilities [17,18] of the ions involved, it is possible to predict quite accurately the index contrast achievable upon laser writing via FLIER [19]. This principle has been successfully applied to several glass families including phosphates and borates where lanthanides have been used as index carriers [4,5] and, more recently, to silicates [8] using Ba as index carrier. 

Apparently, at least in the case of phosphate-based glasses, heavier ions belonging to the same group (i.e., lanthanides (La, Er and Yb)) migrate in the same direction and experience approximately the same amount of enrichment in the higher refractive index region [5]. This apparently applies to the counter-migrating lighter ions (Na, K) too in the low index zone. For the case of Ba, counter-migration of the lighter elements (Na and Si) away from the refractive index increased region has been observed [4,8]. These observations are consistent with what can be called “positive Soret effect” for the heavier constituents of the glass and “negative” for the lighter counter-migrating species, a common observation for thermo-diffusion in glasses or sols. However, not all of the glass constituents necessarily move upon laser excitation. This depends on the material composition [13], the excitation conditions and type of ions involved [20], the shape of the focal volume and the correlated thermal gradients involved [8,21]. 

In the refractive index structures produced by FLIER, ion cross-diffusion occurs in a hot and viscous high-temperature glass matrix. Therefore, one can expect the presence of either cooperative or competition effects when different types of index carriers (belonging to different groups) are present in the glass composition. However, to the best of our knowledge, there are no studies regarding the simultaneous use of several types of index carriers in the production of waveguides by fs-laser writing via FLIER. This aspect is a very relevant for potential applications of fs-laser processing beyond the production of waveguides. As below indicated, FLIER processes can be detrimental for certain properties of multicomponent glasses and, therefore, a deeper knowledge of the migration behavior of different types of ions upon fs-laser excitation is very desirable. In this work, we aim at analyzing the presence of such cooperative or competition ion migration effects upon FLIER in compositionally pre-designed glasses. 

For such a purpose, phosphate glasses modified with the same amount of Na_2_O and K_2_O, and different amounts of BaO and/or La_2_O_3_ were used to produce FLIER-based waveguides. The general behavior and performance of the Ba-only and La-only modified glasses is rather similar in terms of migration threshold, laser energy dependence of the size of the modified index structures and index contrast. However, the samples modified with both Ba and La oxides show a strong increase in the migration threshold, and a clear reduction of the size and index contrast of the structures for comparable writing laser energies. These features indicate that strong migration competition effects occur when index carriers of different types (valence) are introduced in the glass composition. This competition effect can be exploited to inhibit undesired cross-thermo-diffusion effects in fs-laser writing applications in multicomponent glasses where preserving the glass composition may be a requirement. The preservation of the local composition of the glass upon fs-laser irradiation is a key issue for instance in fs-laser-induced crystallization processes for data storage applications [22], the congruent synthesis of non-linear crystals inside precursor glasses by fs-laser writing [23,24] or the fabrication of hybrid micro- and nano-structures in semiconductor-doped glasses by ultrafast lasers [25].

## 2. Materials and Methods 

The phosphate-based glass samples were obtained through melting and quenching of batches made by mixing (NH_4_)_2_HPO_4_, Na_2_CO_3,_ K_2_CO_3_, BaO, and La_2_O_3_ (Sigma-Aldrich Spain, Madrid) reagent grade powders. The compositions of the produced samples are shown in Table 1.

Three different sample compositions (namely Ba-, La- and Ba-La-glass) were produced. In order to facilitate the alignment procedure during the characterization of the guiding performance of the laser-written structures in the infrared (~1600 nm), all the samples were doped with a very small amount of Er_2_O_3_, 0.25 wt.%, and Yb_2_O_3_, 0.5 wt.%, to use the green color up-conversion emission of Er^3+^ when a weak pump laser at 976 nm is coupled in the waveguide [4]. In the Ba-glass sample, a 5 mol% of Al_2_O_3_ was added to the composition in order to improve its toughness and thus being comparable to the one in the La_2_O_3_-containing samples. All glasses were formulated for them to have an O/P ratio close to 3, which in phosphate glasses stands for metaphosphate compositions. The batches were slowly calcined up to 400 °C overnight in alumina crucibles; then, melted at 1000 °C (Ba and Ba-La glasses) or 1200 °C (La-glass) for 1 h. The melts were poured onto brass plates and then crashed into small pieces and put into a graphite boat where they were submitted to a homogenization and fining treatment following the procedure applied for phosphate glasses in [26]. For this case, the glasses were remelted at 800 °C (Ba and Ba-La glasses) and 900 °C (La-glass) for 9 h under a constant flow of N_2_. The final glass samples were completely dehydroxylated and homogenized without any bubbles or stria and no weight loss was detected that could have originated from volatilization losses. The glass transition temperature (T_g_**)** of the samples was determined from thermal expansion curves recorded in a Netzsch 402 EP dilatometer (Netzsch-Gerätebau GmbH, Selb, Germany) at a heating rate of 5 K·min^−1^. The T_g_ of the three glass samples ranged between 510 °C and 550 °C. The observed differences in T_g_ are consistent with the relatively small compositional differences among the samples.

The samples were then cut (typically in the shape of 10 × 20 × 6 mm^3^ plates) and polished to optical quality before optical characterization and fs-laser writing. Ellipsometric spectra were measured at several spatial locations in each sample in order to achieve statistically significant values in the near IR region (800–1700 nm). The acquired Ψ-spectra were fitted using the Cauchy equation ($n\left(\lambda \right)=A+(B/\lambda^{2})$) for the refractive index *n* as a function of the wavelength (*λ*). This dispersion equation describes very well the behavior of the refractive index of the samples in the studied spectral region, where they are transparent (absorption coefficient *k* = 0). Further details regarding the ellipsometry measurement procedure can be found elsewhere [27].

Waveguide-writing via FLIER was performed using a high-repetition-rate, Yb-doped fiber fs-laser (Satsuma HP^2^ laser from Amplitude, Pessac, France). The laser generates laser pulses of tunable duration (350 fs–10 ps) at 1030 nm. The laser has a repetition rate that can be varied in the 1 kHz-2 MHz interval with energies up 10 µJ/pulse. In all the experiments described, we used 350 fs-laser pulses at a repetition rate of 500 kHz. The writing beam was slit-shaped (1.2 mm slit width) before being focused with a 0.68 Numerical Aperture (NA) aspheric lens 100 µm underneath the surface. Its polarization was circular in order to minimize the propagation losses of the waveguides. During writing, the sample was translated at a linear speed of 60 µm/s in all cases. A detailed description of the laser writing setup can be found elsewhere [15,28]. 

Processed samples were grinded and polished to bring the end facets of the laser-written structures to the surface. Optical microscopy observation (in transmission) along the guiding axis of the structures was performed with a Nikon Eclipse system. 

After processing, the near field guided modes of the laser-written structures were also imaged by coupling-in light at several different wavelengths in the 980 nm–1640 nm interval with a SMF-28 single mode fiber and using a 50× objective and an infrared camera (Goodrich SUI). The evaluation of the total losses of each structure was made by measuring the output power of the waveguide at 1640 nm with a power meter (Ophir PD300). Propagation losses were calculated as total minus coupling and Fresnel losses, as described elsewhere [6]. The local elemental composition changes were quantified by using Energy-dispersive X-ray Microanalysis (EDX) in a Scanning Electron Microscope (SEM, Leica Cambridge Ltd., Cambridge, UK).

## 3. Results and Discussion

### 3.1. Optical Properties of the Pre-Designed Glass Samples and Index Change Estimates upon FLIER

Table 2 shows the Cauchy coefficients of the refractive index of the samples obtained from the fit of the ellipsometric parameter Ψ measured at three different spatial locations in each sample. The error in the determination of the refractive index values (absolute) has been estimated to be smaller than $\pm 5\times 10^{-3}$ [27].

**Figure 1:**
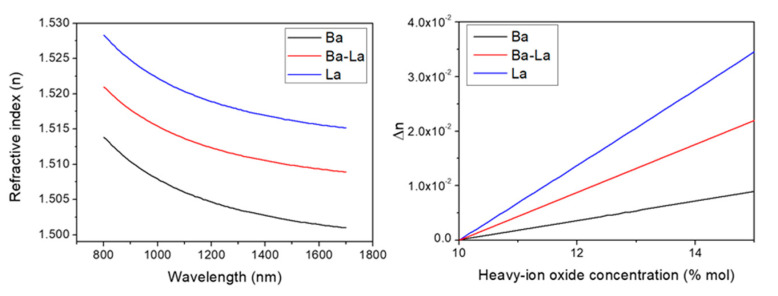
Spectral dependence of the refractive index of the glass samples measured by spectroscopic ellipsometry (**a**). Index change as a function of the heavy-ion concentration estimated by the electronic polarizability method (**b**).

The spectral dependence of the index (*n*) in the 800–1700 nm range has been plotted in Figure 1a for the three glass samples. The measured refractive index values show a very similar dispersion for the three glasses, but the absolute *n* values are distinctly different. The glasses are essentially formed by the same constituents and proportions (~60 mol.% P_2_O_5_ (glass former) and 30 mol.% of Na_2_O and K_2_O (modifiers)). The larger electronic polarizability of La_2_O_3_ compared to BaO provides a larger index to the La-glass over the whole spectral interval, while the Ba-La-glass shows intermediate values also conditioned by the substitution of 5 mol.% of P_2_O_5_ by Al_2_O_3_. The electronic polarizabilities of the constituents of the three glass samples are shown in Table 3.

By using the electronic polarizability method [14,15,29] and the determined refractive index values for wavelengths much longer than the glass matrix resonances, it is possible to accurately estimate the changes in the refractive index of the glass associated to small changes in its local composition caused by FLIER [16]. This approach has been shown to work particularly well in the case of phosphate-based glasses [19,30]. We have used this approach, using the glass constituent’s polarizabilities shown in Table 3, to estimate the index changes that take place when the local concentration of the index carrier oxide increases [19]. For the calculation, it is assumed that the local concentration of the index carrier oxide (BaO or La_2_O_3_) increases by the same amount as the concentration of the fast-diffusing ion oxides (Na_2_O and K_2_O) decreases (e.g., an increase of 2 mol.% of La_2_O_3_ is accompanied by a corresponding decrease of the Na_2_O and K_2_O concentrations of 1 mol.% each). For the two heavy ions considered, the Ba- and La-glass index increases nearly linearly with the local concentration increase of the carrier oxide, due to the relatively minor contribution of the counter-migration of K and Na, with a much smaller polarizability. It is found that the Ba-La glass shows again an intermediate behavior. For a local concentration increase of the index carrier of 20% (relative), index changes above or close to 10^−2^ are expected in the La- and La-Ba-glasses which for the Ba-glass are reduced down to 3.6 × 10^−3^.

### 3.2. Characterization and Performance of the Produced Structures

#### 3.2.1. Morphology of the Laser Written Structures

**Figure 2:**
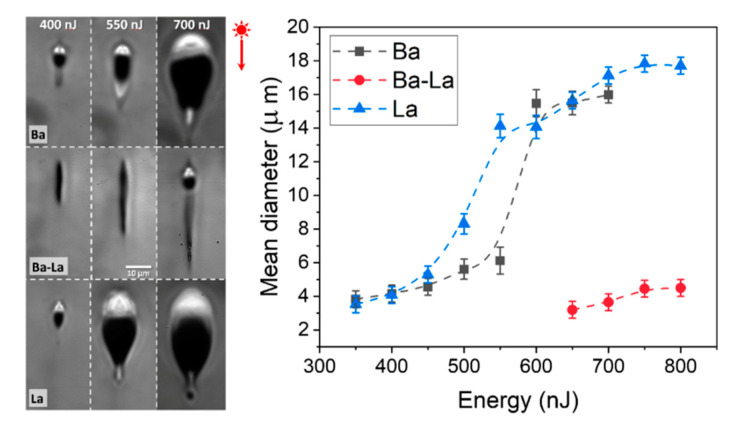
(**Left** panel) Transmission optical microscopy images of the cross section of structures produced in Ba-, Ba-La-and La-samples at the indicated writing pulse energies. (**Right** plot) Mean diameter of the structures produced in the three different composition samples as a function of the writing pulse energy.

Figure 2 shows several optical micrographs in transmission corresponding to the cross section of the laser-written structures transverse to the sample movement axis (*X*) for three representative values of the writing pulse energy. As indicated above, after processing, the samples were polished to bring the end facets of the laser-written structures to the surface and, therefore, the cross sections shown correspond to the buried waveguides produced upon laser writing underneath the surface. A top view of the structures (not shown) indicates that the structures (in the form of a continuous line) homogenously spread along the sample movement axis.

To facilitate their comparison, the images are shown at the same magnification. In all cases, the structures show an elongated shape along the beam propagation axis due to the combined effect of spherical aberration (SA) and non-linear propagation [21,31]. As a matter of fact, the length of the structures along the beam propagation axis is nearly independent of the sample composition for a given pulse energy. This behavior is related to the relatively small compositional differences among the samples, generating relatively small differences in their linear and non-linear refractive indices, that condition, respectively, the extent of SA and non-linear self-focusing experienced by the laser beam. However, the size and shape of the structures in the transverse dimension (perpendicular to both the laser propagation and sample moving axis) show a strong difference between the samples incorporating a single index carrier element (Ba- and La-glass) or two (Ba-La-glass). In the former case, a bright contrasted region with increased refractive index is clearly visible for writing energies above ~350 nJ/pulse. As confirmed by EDX measurements (see below), the high index region is caused by the migration of Ba or La to the top of the structure while the region underneath, depleted in the carrier element, shows a depressed index and shows a dark contrast [4,8,15,27,28]. It is worth noting that Ba or La enrichment can also be observed (to a much lesser extent) at the bottom of the structures produced at the higher energies. This effect has previously been observed both in La- and Ba-modified glasses [4,8,19] and is related, as discussed in Ref. [8], to the actual shape of the generated thermal gradient. The plot in Figure 2 shows the mean diameter of the bright contrasted region at the top of the structures as a function of the writing pulse energy. The mean diameter of the structures was computed as the square root of the product of the dimensions of the bright region along the laser propagation axis (Z) and transversally to it (Y) in the images shown in Figure 2. 

For the Ba- and La-glasses, above ~450 nJ, the size of the structure shows a sudden and strong increase caused by an excessive heat accumulation [32] not compensated by heat diffusion [15]. The earlier increase of the structure size in the La-glass as a function of the laser energy seems thus related to its expectable lower thermal diffusivity compared with that of the Ba-glass. In contrast, in the Ba-La glass the refractive index increased region is observed only for the higher pulse energies studied (above 650 nJ). For lower values, the laser modified zone adopts the appearance of a dark, non-linear self-focusing filament. Such a difference in the material response is also illustrated quantitatively in the plot of Figure 2. While for the Ba- and La- samples, the bright regions are observed for energies as low as 350 nJ, its formation requires ~650 nJ in the Ba-La sample. At this energy and above, its average diameter is ~3–4 µm while the Ba- and La-samples show an average size for the high index zone about four times bigger at a comparable energy. Such morphological differences cannot be attributed to differences in the optical or thermal properties of the Ba-La-glass compared to those of the Ba- and La-glasses, since both optical and thermal properties belong to the group of additive properties of glasses [33] and should therefore show (as seen in the optical case, Figure 1) intermediate values with respect to those observed in the glasses with a “single index carrier”.

The comparison of the morphology of the structures in the three glass compositions analyzed strongly suggests that the laser energy threshold required for generating the cross-migration of the index carrier ions and the alkali species is substantially higher in the Ba-La-sample when compared to the case of the Ba- and La- samples, and that the presence of two heavy index carriers of different valence seems to be hindering their thermo-diffusion against alkali, due to a possible competition effect. In this respect, it is worth noting that a somewhat similar effect of migration inhibition effect has been observed before in the form of two different energy thresholds for inducing the cross-migration of light or heavy ions against alkali in a phosphate glass modified with lanthanides, potassium, silicon and aluminum oxides [20]. The general trend observed there, and later on quantitatively confirmed in [5], was that all the lanthanides showed a similar migration threshold and moved by the same extent and in the same direction. Above such a threshold, the migration of the lighter non-alkali elements was apparently inhibited or minimized below the EDX measurements resolution [20]. 

#### 3.2.2. Guiding Performance of the Structures

**Figure 3:**
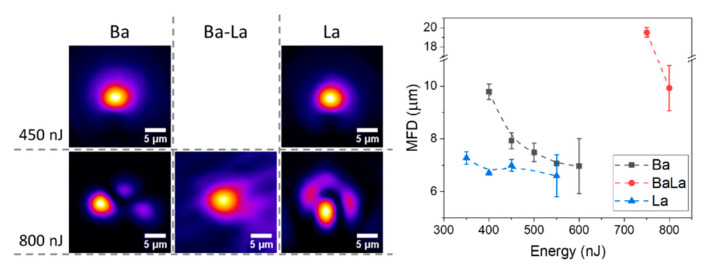
(**Left** panel) Near field images and (**right** plot) corresponding mode field diameters (MFD) at 1590 nm for the single-mode structures produced in the different glass compositions.

The performance as optical waveguides of the compositional structures generated via FLIER in the different glass samples was assessed for laser wavelengths in the 980 nm–1640 nm interval. Figure 3 shows the near field image of the propagated modes at 1590 nm for two representative laser pulse energies in the three glass samples. As anticipated from the morphology data, the structures produced in the Ba-La-glass do not support any guided modes for energies below 700 nJ. On the contrary, the structures produced in Ba- and La-glasses show single mode propagation and a relatively small mode field diameter (MFD) for energies between ~400 nJ up to approximately 600 nJ where they start to show multimodal propagation.

The plot in Figure 3 shows the evolution of the MFD (for the single mode waveguides) as a function of the laser pulse energy. The plot indicates that the waveguide formation threshold is slightly smaller for the La-glass (350 nJ) while the MFD remains nearly constant (~7 µm) for pulse energies up to ~550 nJ. At this energy, the transmission of the waveguides turns multimodal, generating a large uncertainty in the MFD estimation. For the Ba-glass, at its waveguide formation threshold (400 nJ), the MFD (~10 µm) is substantially larger than for the La-glass and decreases with the pulse energy down to values close to 7 µm. As for the La-glass, above a certain value (600 nJ), the structures behave as multimodal. 

**Figure 4:**
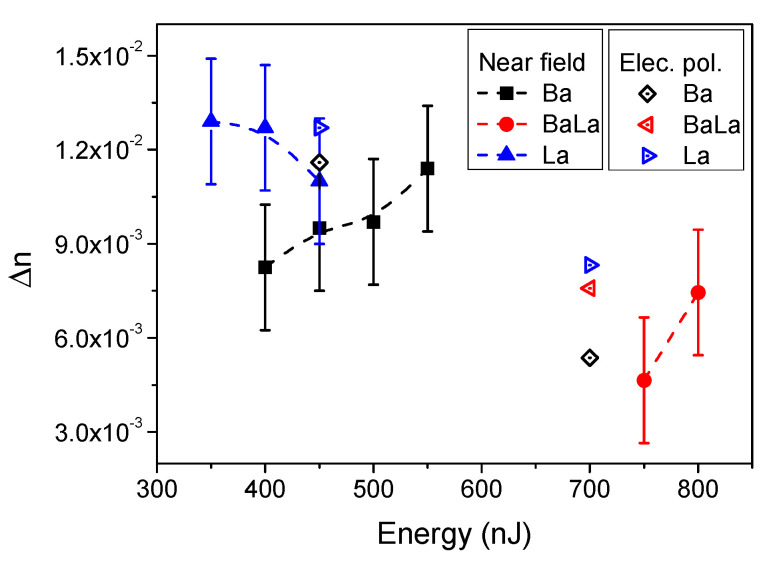
Refractive index contrast at the guiding region estimated from the measured MFD of the single mode waveguides produced in the three glass compositions indicated (Ba-, La- and Ba-La-glass) (solid symbols). The open symbols correspond to the index contrast estimated using experimental EDX local compositional measurements and the electronic polarizability method, as described in the text.

The differences observed as a function of laser pulse energy in the behavior of the waveguides written in the Ba- and La-glasses are consistent with the expected differences in their thermal diffusivity and in the polarizabilities of the index carriers involved. The lower diffusivity of the La-glass and the higher polarizability of La_2_O_3_ facilitates the formation of a structure capable of supporting a single guide mode for energies slightly lower than those required for the he Ba-glass. However, in the latter case, at the waveguide formation threshold, the index increase in the guiding region is clearly smaller than in the La-glass (larger MFD for the same structure size, Figure 2) due to the lower index change expected upon Ba-enrichment. For the same amount of local enrichment of the index carrier (Ba or La), the local index increase should be about three times larger for the La-glass. This can be more clearly seen in Figure 4, where we have plotted the index contrast of the single mode structures as a function of the writing pulse energy, as derived from the size of the high index region and its mode field diameter for several wavelengths [19,34]. The figure shows that, already at the waveguide formation threshold, an index contrast above 10^−2^ is induced in the La-glass. This parameter shows a nearly constant behavior in the single mode region (350–550 nJ/pulse). According to the plot in Figure 2, for energies in this interval, the index contrast observed is consistent with a local enrichment in La in the guiding region around and above 20% relative to the initial material composition. This might suggest that the transition to a multimodal behavior in the La-glass sample above 550 nJ might be rather conditioned by the size of the structure than by the index contrast achieved, at least in the vicinity of the multimodal transition energy at ~550 nJ.

The index contrast of the Ba-glass sample shows nevertheless an increasing behavior with the laser pulse energy. Given the slower increase of the structure size with energy in this case, and the clear diminution of the MFD with the same parameter, the observed behavior suggests a progressive increase in the local content of Ba in the guiding region, that can lead to index contrast values above 10^−2^. By using the values in Figure 2, a local Ba relative enrichment above 40% is foreseen.

Once again, the behavior of the laser-written structures in the Ba-La-glass strongly differs and is not “in between” that observed for the Ba- and La-glass samples. Along with a waveguide formation threshold about twice as big (750 nJ), the index contrast achieved in the guiding region is much smaller, about a factor of two smaller than the one in La-glass. This comparatively poor contrast would be consistent (see Figure 2) with a local enrichment around 10% in the index carriers, much smaller than that observed in the Ba- and La-samples at a much higher laser writing energy. This aspect points again to the presence of strong migration competition effects that are even more evident in the measured local compositional changes described below.

Regarding the propagation losses, although the optimization of this parameter is beyond the scope of this work, it must be emphasized that the propagation losses achieved in the structures compare well with previous results of FLIER waveguides based on phosphate- and silicate-based glasses for the Ba- and La-glass samples [4,5,6,8]. In the La-glass sample, the losses in the single mode structures are typically in the 0.5–1.0 dB/cm range, while, in the Ba-glass samples, they are somewhat higher, in the 0.7–1.3 dB/cm interval. Much higher losses (2–3 dB/cm) are observed in the structures produced in the Ba-La glass samples. This can be related to both the lower index contrast and much higher writing energies, causing energy coupling fluctuations.

#### 3.2.3. Local Compositional Changes and Migration Competition Effects

**Figure 5:**
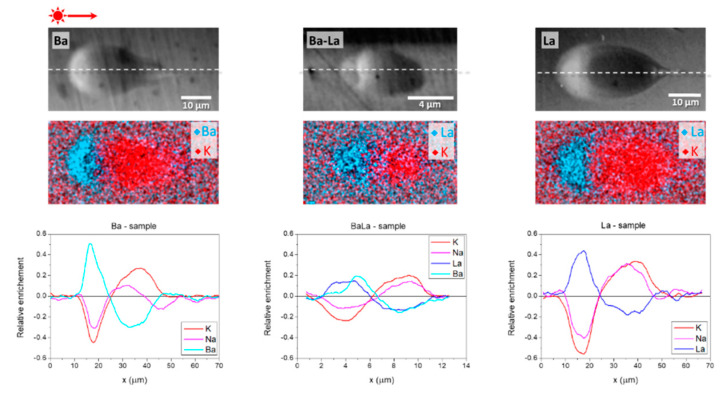
(**Top** row) SEM backscattered electron images and EDX compositional maps corresponding to Ba, La, and K for structures produced at 700 nJ in the Ba-, La- and Ba-La-glass samples. (**Bottom** row) Corresponding composition line scans along the dashed line shown in the SEM images.

As indicated in Section 2, the local composition of the laser-induced structures was measured in an SEM by means of EDX. Figure 5 shows several representative SEM images and compositional maps of structures produced at the same energy in the three samples (700 nJ). Since the images have been recorded on the cross sections of the structures normal to the sample scan axis, they can be easily compared with those shown in Figure 2. For all of them, the enrichment in the heavier elements in the guiding region is evidenced by the increased Z-contrast (bright zones) in the backscattered electron SEM images. The correspondence between optical and Z-contrast in FLIER-based optical waveguides has been widely reported (see for instance [4,6,15]). The opposite contrast (dark contrasted zones) is consistent with a local depletion of heavy elements, leading to depressed index zone adjacent to the guiding region.

This can be more clearly appreciated in the compositional maps included, corresponding to the spatial distribution of La, Ba and K in each of the samples (for clarity, the spatial distribution of Na is not included in the maps but is shown in the compositional cross sections in the same figure). For the selected energy (700 nJ), the structures in the Ba- and La-glass show very similar appearance including the similarities in the size and element distribution maps (obviously replacing Ba by La in the corresponding sample). As above indicated, both structures are multimode while for the Ba-La glass the structure shown, just above the FLIER threshold (650 nJ in this sample) does not support a guided mode.

Several additional features are worth noting in the element distribution over the FLIER region. The first one is that the relative enrichment in Ba and La, respectively, in the guiding region of the Ba- and La-glass structures reaches a very similar value, close to 50%, slightly higher in the Ba-glass case. This implies that the guiding region reaches a peak concentration in the index carrier oxide close to 15 mol% in the guiding region. Such an enrichment is accompanied by a more or less similar relative depletion in K and Na, although the K depletion is always bigger than the Na one. The same applies to the longer region depleted in Ba or La, located below the guiding zone, with a depletion around 20–30%, slightly higher in the Ba case. Interestingly, the extent of the region over which compositional changes are observed is also in both glass samples somewhat longer for the K than for the Na case.

The index contrast estimates derived from the structure size (see Figure 2) and MFD measurements (see Figure 3) have been compared to the values derived from the local composition of the structures obtained by the EDX and the electronic polarizability approach, as described in [19]. The index contrast was computed by using an averaged composition over the index carrier enrichment region by integrating the compositional cross sections of each individual element except oxygen (see Figure 5) and assuming oxide stoichiometry. The so-calculated averaged composition was input in the electronic polarizabilities model to compute the averaged local index for two energies (450 and 700 nJ). The obtained values have been plotted in Figure 4. It can be seen that there is an excellent correlation between the measured compositional changes and the experimentally determined index contrast in the single mode region where Δ*n* values close to 1.2 × 10^−2^ are reached for both the Ba- and La-glass samples. At energies above 600 nJ, the index contrast estimated from compositional measurements decreases to values ~7.0 × 10^−3^, something expectable from the much bigger size of the structure over which the index carrier is redistributed, also causing the observed multimodal behavior.

In contrast, in the Ba-La-glass sample the relative enrichment in Ba- and La- at the top of the structure at 700 nJ is much smaller, around 15–20% (less than half of what is observed in the Ba- and La-glass samples, compare the linescans shown in Figure 5). Since the initial content of the sample in La_2_O_3_ and BaO is just 5 mol%, the peak concentration of index carrier oxides barely reaches 12 mol%, much lower than in the Ba- or La-glass samples at the same energy. The same applies to the Na and K depletion, that is lower than 20% in average. It is also worth noting that the amount of enrichment and depletion in the Ba- and La- content is similar in the two regions of the structure (bright and dark-contrasted in the SEM image), generating a nearly symmetric compositional structure along the laser beam propagation axis. Indeed, the overall length of the compositionally modified FLIER region in the Ba-La glass (~10 µm) is much smaller than the one in the Ba- and La-glass samples at the same energy (~60 µm). Notice that the length of the laser-affected zone along the laser beam propagation axis is very similar for the three glass compositions, at the same laser pulse energy, as shown in Figure 2, and longer than the extension of the FLIER region. The index contrast estimate based on EDX compositional measurements at 700 nJ leads to a value around 5 × 10^−3^ which is consistent with the fact that the structure, less than ~4 µm in diameter, does not support a guided mode in the near IR.

All these features of the structures produced in the Ba-La-glass, compared to the behavior of the Ba- and La-glass samples, show that the cross-migration of Ba and La against alkalis is hindered by the simultaneous presence of the two index carriers. Whatever the origin of this behavior is, the presence of both (Ba and La) oxide modifiers clearly inhibits their cross-migration against the alkalis over a very broad interval of laser pulse energies (more than 700 nJ) while, above the cross-migration threshold, it reduces the extent of the migration process both in magnitude (smaller compositional changes) and spatial spread (shorter compositionally modified regions).

This kind of blocking effect somewhat resembles the so-called mixed alkali effect (MAE) in glasses [35,36,37], although in a very different situation. Please note that in what follows in the comparison with the MAE, we refer not to the presence of Na_2_O and K_2_O as fast diffusing modifiers in the glass compositions. In this case, we refer to the concurrent effect associated with the presence of both index carriers (Ba and La) that hinders their cross-thermo-diffusion against the alkalis in the FLIER process.

In general, the MAE refers to several phenomena peculiar to mixed-cation glasses, including the huge rise in electrical resistivity occurring on mixing of two mobile ions [35]. Indeed, although the MAE, evidenced as a non-additivity of many glass properties, was initially observed as a thermomechanical effect, it has been more thoroughly described in terms of the decrease of several orders of magnitude in the electrical conductivity and ionic diffusivity when one alkali oxide is gradually replaced by another in a series of glasses [36,37]. Such a decrease is accompanied by a strong increase of the activation energy of the ionic conductivity [35]. It is also worth noting that the MAE has been observed in a wide variety of glass families, including silicate, borate and phosphate-based glasses, something that can be related to the fact that alkalis are usually considered to simply fill the interstitial space between structural rigid polyhedra and convert bridging oxygens to nonbridging oxygens [37].

The origin of the MAE has been correlated with the interaction between the alkali cations via structural defects such as non-bridging oxygens [38], “memory effects” [39,40] or, more recently, to topological effects caused by the network strain associated to different cation radii [37]. An important common feature of these “memory” or topological effects is that alkali ions have individualized sites that relax to suit their inhabitants below T_g_. According to the thorough literature summary given in [37], “when the alkali leaves the site, the site retains “memory” of that ion, making it difficult for ions of the other type to hop into it”. The recent use of detailed molecular dynamics (MD) simulations and comparison to MAE experiments enables to confirm that interstices containing mismatched alkali species relax toward different ultimate structures and the competition between these interstices results in stress on the network [38].

When comparing our results regarding the cross-migration of Ba and La ions against alkali by thermo-diffusion in the Ba-, La-, and Ba-La-glass samples, there are two features that strongly resemble the MAE. The first is the existence of an energy threshold for the cross-diffusion of species in the FLIER process that resembles the existence of an activation energy for the ionic conductivity in fast ionic conductors [41], directly related to the diffusion of cations in the presence of a DC electric field. The second is the strong increase in the cross thermo-diffusion threshold in terms of laser pulse energy when both Ba- and La- oxides are present in the composition, as similarly observed with the ionic diffusion activation energy in the MAE. The atomic number, mass, valence, and ionic radii of the ions involved in the FLIER process are shown in Table 4.

The table shows that, in spite of their very close atomic number and mass, there is a relatively large difference between the cationic effective radii of Ba^+2^ and La^+3^ (~20%). We might speculate that, even at high temperature, for a low-viscosity glass material that is generated upon high repetition (500 kHz) pulsed laser irradiation, there is still a “memory effect” associated with the type of ion that occupied a given site in the glass. In such a case, we may think of a “memory effect” like the one described in the frame of the dynamic structure model [39,40] or like the one associated with the network strain model [37]. Whatsoever the origin of the “memory effect” is, this would imply that pathways for ion thermo-diffusion are limited to sites of that ion type, and are dependent on relaxation of those sites in order to form new diffusion pathways. The presence of a cation in an unlike site would be increasingly less likely to form with an increasing size ratio [37]. The consequence would be that in the Ba-La glass sample, the sites left by the jump of a given heavy cation would most probably be used by an index carrier of the same type, hindering the overall cross-migration process.

However, when several lanthanides are present in the composition of a phosphate-based glass, they experience similar levels of thermo-diffusion in the same direction, as experimentally shown in references [5,20] for La, Er and Yb with the same valence (+3), and ionic radii differing around 10%. Therefore, despite the cation size may play a certain role in limiting the simultaneous diffusion of index carrier elements, it seems that it would not be sufficient by itself to account for the high differences here observed between the Ba- or La-glass samples and the Ba-La-glass. Additionally, it must be considered that the MAE is strongly reduced when the temperature of the glass increases, as it has been observed for the thermal conductivity or the viscosity of phosphate and aluminosilicate glasses [42,43,44], which makes it difficult to justify the existence of a similar effect in a very high temperature melt, such as the one induced upon fs-laser irradiation.

Besides the size (ionic radius) of the element, there is an additional factor that may turn out decisive and that has not been considered before, which is the valence of the index carrier elements. When having single valence index carrier elements, e.g., lanthanides as indicated above, their cross-diffusion with the alkalis takes place all at once without mutual interference, even for those with relatively dissimilar size. At the temperatures involved in the process, the relaxation of the network will be sufficiently fast so that cations with different size but equal valence migrate without constraints. However, the cross-migration of index carrier cations would also be regulated through the electrostatic interaction with the electronic density of the oxygens that belong to the PO_4_ network building units. Same valence elements can cross-diffuse with alkalis in order to easily keep the charge neutrality. This makes that Ba^2+^ ions only migrate through positions of nearby Ba^2+^ and that La^3+^ do so via La^3+^ positions to not produce local decompensated charges, resulting in a largely reduced cross-migration. Similarly, to the mixed modifier effect that accounts for non-linear property changes in the presence of different size cations, it is proposed that here applies what could be called a “mixed valence effect” and that would be worth validating in other glass composition systems. Whatsoever the origin of the migration competition effects is, they could potentially be used to inhibit thermo-migration effects in fs-laser writing applications where the preservation of the composition of the glass can be needed as a requirement [13,20,22,23,24,25].

## 4. Conclusions

We have analyzed the formation of refractive index micro-structures by FLIER in ad hoc compositionally designed phosphate glasses. The glasses were modified by the addition of small amounts of Na_2_O and K_2_O and heavier ion (Ba and La) oxides. The latter ions act as index carriers. As a consequence of the laser excitation, cross-thermo-diffusion of the alkalis and index carrier cations occurs in the excited volume. Regions enriched in BaO and La_2_O_3_ and depleted in N_2_O and K_2_O are formed in the laser-excited volume. These regions show an increased refractive index sufficient to support guided modes in the near IR. The glass samples modified with a single index carrier ion oxide (either Ba- or La-oxide) show a relatively low waveguide writing threshold (350–400 nJ) and a light guiding performance comparable to each other. In both cases, index contrast values above 10^−2^, and low propagation losses are achieved. In contrast, when both types of index carriers (Ba and La cations) are present in the glass composition, a much higher element migration threshold is observed, and the size of the compositionally modified regions is substantially smaller. Indeed, the cross-thermo-diffusion of Ba and La against Na and K is inhibited for laser pulse energies below 750 nJ. The morphological, optical and compositional analysis of the produced structures is consistent with a competition effect in the thermo-diffusion of Ba^2+^ and La^3+^ cations that strongly resembles the mixed alkali effect (MAE). Such a diffusion competition effect, apparently related to different valence of the index carrier ions involved, provides a feasible compositional strategy to suppress FLIER effects. This compositional design approach can be crucial to enable the use of fs-laser processing in multicomponent glasses where the preservation of the local composition of the glass is a necessary requirement to preserve some specific properties of the glass.

## Figures and Tables

**Table 1:** Composition of the designed samples.

Sample	Alkali Oxide Modifiers		Refractive Index Carrier Modifier		Glass Former	Other Modifiers
	Oxides Composition in mol %					
	K_2_O	Na_2_O	BaO	La_2_O_3_	P_2_O_5_	Al_2_O_3_
Ba-glass	15	15	10	0	55	5
La-glass	15	15	0	10	60	0
Ba-La-glass	15	15	5	5	60	0

**Table 2:** Cauchy coefficients of the samples before laser processing and index values at 1590 nm.

Sample	Cauchy Coefficients		Refractive Index (1590 nm)
	A	B	
Ba-glass	1.114	0.0141	1.500
La-glass	1.133	0.0144	1.515
Ba-La-glass	1.125	0.0133	1.510

**Table 3:** Polarizabilities of ions used for local index calculations in the samples upon FLIER.

Polarizability (Å^3^)	P_2_O_5_	Na_2_O	K_2_O	BaO	La_2_O_3_	Al_2_O_3_
$\alpha_{cation}$	0.021	0.175	0.841	1.595	1.048	0.054
$\alpha_{O}^{2-}$	1.350	3.221	1.858	3.652	2.780	1.365
$\alpha_{Total}$	6.792	3.571	3.540	5.247	10.436	4.203

**Table 4:** Atomic number, mass valence and ionic radius (crystalline and effective, assuming a coordination number of six [36]) of the cations involved in the FLIER process.

Element	Na	K	Ba	La
Atomic number	11	19	56	57
Mass (a.m.u.)	22.99	39.10	137.3	138.9
Valence	+1	+1	+2	+3
Cation radius (pm) Crystalline (Effective)	116 (102)	152 (138)	140 (135)	117.2 (103.2)

## Data Availability

The data presented in this study are available on request from the corresponding authors.
